# Cytotoxicity and Genotoxicity of Nanomaterials

**DOI:** 10.3390/nano12040634

**Published:** 2022-02-14

**Authors:** Vanessa Valdiglesias

**Affiliations:** 1 Grupo NanoToxGen, Centro de Investigacións Científicas Avanzadas (CICA), Departamento de Biología, Universidade da Coruña, 15071 A Coruña, Spain; vvaldiglesias@udc.es; 2 Instituto de Investigación Biomédica de A Coruña (INIBIC), Oza, 15071 A Coruña, Spain

Nanomaterials (NMs) are of significant relevance due to their unique physicochemical properties, which have been extensively exploited for widespread applications in human healthcare and consumer goods, such as cosmetics and textiles. They are also being explored for an emerging number of bio-applications, particularly in medicine and the pharmacology industry, for drug delivery, therapeutics and diagnosis. As a result, human exposure to these NM, both occupationally or environmentally, is increasing rapidly, becoming an issue of concern to public health because they are able to reach the bloodstream by efficiently crossing membrane barriers, are distributed throughout the whole body and exert effects in organs and tissues at cellular and molecular levels [1]. In this frame, approaches aimed at revealing or discarding the possible toxicity of NMs, particularly those at these levels, are essential to understand the potential risks to human health associated with their exposure, and to ensure the proper regulation of the production and use of these materials.

Nevertheless, there is still limited knowledge on the potential side effects and toxicity profile of most NMs, which leads to a lack of adequate regulation for testing and the safe use of NMs in industry and medicine. This is due in part to the singular physicochemical features of NM make it particularly difficult to explore their behavior on biological systems, but also because the most widespread toxicological methodologies and testing guidelines commonly used to assess the possible harmfulness of different chemical agents are not fully adequate to be applied to NMs [2,3]. These shortcomings slow down the progress in develop nanotoxicology knowledge; consequently, the NM effects on both the environment and human health remain largely unknown.

On this basis, and given the prime importance of defining mechanisms for nanotoxicity evaluation and the urgency of establishing conditions for the safety use of NMs, this Special Issue, entitled “Cytotoxicity and Genotoxicity of Nanomaterials”, includes a number of studies aimed at improving the current knowledge on the topic by determining the effects of different NMs, including both 2D and nanoparticles, on a number of in vitro and in vivo systems. In particular, several genotoxic and cytotoxic effects were evaluated and found to be associated with nanoparticle exposure. Related topics, such as the relevance of NM properties and experimental conditions [4,5,6], or the adequacy of the traditional genotoxicity approaches for NP testing [7], are also addressed in this Special Issue.

Graphene and graphene-based NMs have attracted the most attention in nanotechnology industry because of their unique physical, chemical, and mechanical properties. They have been also widely investigated for biomedical applications due to their exceptional qualities, including two-dimensional planar structure, wide surface area, chemical and mechanical constancy, sublime conductivity and excellent biocompatibility [8]. This Special Issue addresses, among others, the potential toxicity of some of these NMs. Particularly, the interactions of graphene oxide (GO) and reduced graphene oxide (rGO) with different polymeric dispersants, such as glycol chitosan, propylene glycol alginate, and polydopamine, as well as their effects on human chondrocytes, have been explored [9]. Cytotoxic effects induced by coated and uncoated GO and rGO on human chondrocytes were assessed through lactate dehydrogenase (LDH) assays. Results showed a concentration-dependent response, and the presence of PGA contributed to significantly decreasing the difference in LDH activity with respect to the control. Kostyuk et al. [4] reported that the chemical structure of fullerenes, another type of carbon-based NM, seems to be decisive in their cytoprotective or cytotoxic properties; some types of functionalized fullerenes are able to regulate the intracellular reactive oxygen species homeostasis and to enhance the antioxidant capacity of human fetal lung fibroblasts. Accordingly, another study demonstrated the lack of cellular effects after exposure to water-soluble glycofullerene (Sweet-C60), suggesting the potential of these NMs to be used as a drug delivery vehicle for treating pancreatic cancer [10].

Additionally, metal and metal oxide nanoparticles (NPs) have gained particular interest in nanotechnology industry. They are often used as industrial catalysts or to improve product functional properties, becoming the most frequent NM present in consumer products. The widescale use of these NPs in the global consumer market has resulted in increases in the likelihood of exposure to human beings. Assessment of the potential side effects of some of the most commonly used metal oxide NP, i.e., cerium dioxide, zinc oxide, titanium dioxide, iron oxide and silica NP, on different biological systems, is also included in this Special Issue. In vitro studies evaluating potential nanotoxicity in human primary cells showed that titanium dioxide (TiO_2_), zinc oxide (ZnO), and cerium dioxide (CeO_2_) NP induced both primary and oxidative DNA damage in salivary leukocytes, which were proved to be a suitable biomatrix for nanogenotoxicity studies using the comet assay [7]. Nevertheless, ZnO NPs were found not to produce immune modulations in exposed peripheral blood leukocytes [6]. In addition, employing different in vitro models, García-Salvador et al. [11] demonstrated the minor toxicity of CeO_2_ NP in acute exposure, but cytotoxic and inflammatory responses in subchronic exposures (60 days), suggesting the need for performing subchronic toxicological studies in order to complete acute toxicity studies and accurately assess the toxicity of NMs and their cumulative effects in organisms. For the first time, Stan et al. [12] explored the molecular mechanisms involved in in vitro pulmonary cytotoxicity triggered by the long-term exposure to silicon-based quantum dots. Exposure to these nanomaterials reduced cell viability and induced increases in the expression levels of several proteins, including P53, the apoptosis-inducing factor, and the autophagy-related proteins Beclin-1 and LC-3, suggesting that the presence of these NPs triggered the activation of apoptotic and autophagy pathways, and the downregulation of survival signaling molecules as an adaptive response to cellular stress. Similarly, Coccini et al. [13] found a reduction in neuronal differentiation and cell mortality in a dose-dependent manner in primary neuronal-like cells, a human stem-cell-derived in vitro model, after magnetite NP exposure.

In vivo studies evaluating the potential harmful effects of NP exposure are also included in the Special Issue. Different variants of amorphous silica nanomaterial (aSiO_2_ NM) were found to induce oxidative DNA damage and DNA strand breaks in the lung cells of rat exposed by inhalation [14]. Nie et al. [15] reported that hepatotoxicity was induced by TiO_2_ NPs in young orally exposed rats, and demonstrated the antioxidant effect of *Lactobacillus rhamnosus* on that NP-induced hepatotoxicity.

In summary, the results obtained from the studies collected in this Special Issue proved the toxic effects of several types of NM and the major influence that the physicochemical characteristics exert on these effects. Together with increasing the current knowledge on the toxicological profile of these particular NPs, these studies set the basis for future investigations in this line, highlighting the challenges in nanotoxicity assessments; mainly the ability of NPs to interfere with standard toxicity assays, the influence of the physicochemical features, as well as the experimental conditions on the observed effects, and the need for performing simultaneous and different approaches to provide more reliable data.

## References

[1] Gubala V., Johnston L.J., Krug H.F., Moore C.J., Ober C.K., Schwenk M., Vert M. (2018). Engineered nanomaterials and human health: Part 2. Applications and nanotoxicology (IUPAC Technical Report). Pure Appl. Chem..

[2] Costa C., Brandão F., Bessa M.J., Costa S., Valdiglesias V., Kiliç G., Fernández-Bertólez N., Quaresma P., Pereira E., Pásaro E. (2016). In vitro cytotoxicity of superparamagnetic iron oxide nanoparticles on neuronal and glial cells. Evaluation of nanoparticle interference with viability tests. J. Appl. Toxicol..

[3] Fernández-Bertólez N., Brandão F., Costa C., Pásaro E., Teixeira J., Laffon B., Valdiglesias V. (2021). Suitability of the In Vitro Cytokinesis-Block Micronucleus Test for Genotoxicity Assessment of TiO_2_ Nanoparticles on SH-SY5Y Cells. Int. J. Mol. Sci..

[4] Kostyuk S.V., Proskurnina E.V., Savinova E.A., Ershova E.S., Kraevaya O.A., Kameneva L.V., Umryukhin P.E., Dolgikh O.A., Kutsev S.I., Troshin P.A. (2020). Effects of Functionalized Fullerenes on ROS Homeostasis Determine Their Cytoprotective or Cytotoxic Properties. Nanomaterials.

[5] Rueda-Gensini L., Cifuentes J., Castellanos M.C., Puentes P.R., Serna J.A., Muñoz-Camargo C., Cruz J.C. (2020). Tailoring Iron Oxide Nanoparticles for Efficient Cellular Internalization and Endosomal Escape. Nanomaterials.

[6] Moratin H., Ickrath P., Scherzad A., Meyer T.J., Naczenski S., Hagen R., Hackenberg S. (2021). Investigation of the Immune Modulatory Potential of Zinc Oxide Nanoparticles in Human Lymphocytes. Nanomaterials.

[7] Valdiglesias V., Fernández-Bertólez N., Lema-Arranz C., Rodríguez-Fernández R., Pásaro E., Reis A.T., Teixeira J.P., Costa C., Laffon B. (2021). Salivary Leucocytes as In Vitro Model to Evaluate Nanoparticle-Induced DNA Damage. Nanomaterials.

[8] Daniyal M., Liu B., Wang W. (2020). Comprehensive Review on Graphene Oxide for Use in Drug Delivery System. Curr. Med. Chem..

[9] Vannozzi L., Catalano E., Telkhozhayeva M., Teblum E., Yarmolenko A., Avraham E.S., Konar R., Nessim G.D., Ricotti L. (2021). Graphene Oxide and Reduced Graphene Oxide Nanoflakes Coated with Glycol Chitosan, Propylene Glycol Alginate, and Polydopamine: Characterization and Cytotoxicity in Human Chondrocytes. Nanomaterials.

[10] Barańska E., Wiecheć-Cudak O., Rak M., Bienia A., Mrozek-Wilczkiewicz A., Krzykawska-Serda M., Serda M. (2021). Interactions of a Water-Soluble Glycofullerene with Glucose Transporter 1. Analysis of the Cellular Effects on a Pancreatic Tumor Model. Nanomaterials.

[11] García-Salvador A., Katsumiti A., Rojas E., Aristimuño C., Betanzos M., Martínez-Moro M., Moya S.E., Goñi-de-Cerio F. (2021). A Complete In Vitro Toxicological Assessment of the Biological Effects of Cerium Oxide Nanoparticles: From Acute Toxicity to Multi-Dose Subchronic Cytotoxicity Study. Nanomaterials.

[12] Stan M.S., Badea S., Hermenean A., Herman H., Trica B., Sbarcea B.G., Dinischiotu A. (2021). New Insights into the Cell Death Signaling Pathways Triggered by Long-Term Exposure to Silicon-Based Quantum Dots in Human Lung Fibroblasts. Nanomaterials.

[13] Coccini T., Pignatti P., Spinillo A., De Simone U. (2020). Developmental Neurotoxicity Screening for Nanoparticles Using Neuron-Like Cells of Human Umbilical Cord Mesenchymal Stem Cells: Example with Magnetite Nanoparticles. Nanomaterials.

[14] Brandão F., Costa C., Bessa M.J., Dumortier E., Debacq-Chainiaux F., Hubaux R., Salmon M., Laloy J., Stan M.S., Hermenean A. (2021). Genotoxicity and Gene Expression in the Rat Lung Tissue following Instillation and Inhalation of Different Variants of Amorphous Silica Nanomaterials (aSiO_2_ NM). Nanomaterials.

[15] Nie P., Wang M., Zhao Y., Liu S., Chen L., Xu H. (2021). Protective Effect of *Lactobacillus rhamnosus* GG on TiO_2_ Nanoparticles-Induced Oxidative Stress Damage in the Liver of Young Rats. Nanomaterials.

